# Delay Differential Model for Tumour-Immune Response with Chemoimmunotherapy and Optimal Control

**DOI:** 10.1155/2014/982978

**Published:** 2014-08-14

**Authors:** F. A. Rihan, D. H. Abdelrahman, F. Al-Maskari, F. Ibrahim, M. A. Abdeen

**Affiliations:** ^1^Department of Mathematical Sciences, College of Science, UAE University, P.O. Box 15551, Al-Ain, UAE; ^2^Department of Mathematics, Faculty of Science, Helwan University, Cairo 11795, Egypt; ^3^Zayed bin Sultan Al Nahyan Center for Health Sciences, College of Medicine and Health Sciences, UAE University, P.O. Box 17666, Al-Ain, UAE; ^4^Institut für Angewandte Mathematik, LS III, TU Dortmund, Vogelpothsweg 87, 44227 Dortmund, Germany; ^5^Department of Mathematics, Faculty of Science, South Valley University, Qena 83523, Egypt

## Abstract

We present a delay differential model with optimal control that describes the interactions of the tumour cells and immune response cells with external therapy. The intracellular delay is incorporated into the model to justify the time required to stimulate the effector cells. The optimal control variables are incorporated to identify the best treatment strategy with minimum side effects by blocking the production of new tumour cells and keeping the number of normal cells above 75% of its carrying capacity. Existence of the optimal control pair and optimality system are established. Pontryagin's maximum principle is applicable to characterize the optimal controls. The model displays a tumour-free steady state and up to three coexisting steady states. The numerical results show that the optimal treatment strategies reduce the tumour cells load and increase the effector cells after a few days of therapy. The performance of combination therapy protocol of immunochemotherapy is better than the standard protocol of chemotherapy alone.

## 1. Introduction

Cancer is considered one of the most complicated diseases to be treated clinically and one of the main causes of death. Accordingly, a great research effort is being devoted to understand the interaction between the tumour cells and the immune system. The treatment of cancer is then one of the most challenging problems of modern medicine. The cancer treatment should kill cancer cells in the entire body and in the meantime keep the healthy cells above the minimum level. Chemotherapy is one of the most prominent cancer treatment modalities. However, it is not always a comprehensive solution for tumor regression. Other treatment options, including surgery, immunotherapy, and radiation, are often able to force the cancer into remission, but better and suitable treatments are needed to fulfil the requirements [1–3].

Recently, chemotherapy is used in combination with immunotherapy to protect the patient from opportunistic infection, as well as fighting the cancer itself [4, 5]. This is due to the fact that the chemotherapy treatment kills both cancerous and healthy cells and therefore it depletes the patients immune system, making the patient prone to dangerous infections. For this and other reasons, it is desirable to strengthen the immune system after an immune-depleting course of chemotherapy. Additionally, however, the ability to recruit the body's own defenses to fight the cancer can be a powerful treatment strategy. Therefore, maintaining a strong immune system, by combining immunotherapy during chemotherapy, may be essential to successfully fight the cancer. However, the query now is how to most effectively combine cancer immunotherapy and chemotherapy?

Mathematical models, using ordinary, partial, and delay differential equations, play an important role in understanding the dynamics and tracking tumour and immune populations over time (see, e.g., [6–14]). Kuznetsov et al. [3] model the interactions of cytotoxic T lymphocyte (CTL) response and the growth of an immunogenic tumour. In the contributions of [15–17], the authors take into account the penetration of the tumour cells by the effector cells, which simultaneously causes the inactivation of effector cells. Recently, in [18], the authors adopted a predator-prey formulation of the tumour immunity problem as a battle between immune cells and tumour cells (predators and prey, resp.). Many research papers have also been done on formulations of the mathematical models describing the interaction between tumour cells and immune cells alone, between tumour cells and normal cells alone, and between tumour cells and chemotherapy treatment alone [19, 20]. We should mention here that the application of the optimal control theory requires the boundedness of the solutions of the model populations; see also [21, 22].

The objective of this paper is to adopt a delay differential model and analyze and provide computationally an optimal way to combine chemotherapy and immunotherapy treatment strategies that identify the best treatment strategy that can minimize the tumor load while maximizing the strength of the immune system. We formulate and analyze a delay differential model describing immune response and tumour cells under the influence of chemotherapy alone and the combinations of chemotherapy and immunotherapy. The outline of the paper is as follows. In Section 2, we describe the model. In Section 3, we study the qualitative behaviour of the model without external therapy. In Section 4, we describe the optimal control problem governed by DDEs with only chemotherapy control variable. Existence of the solution and optimality conditions are discussed in Sections 5 and 6. In Section 7, we extend the control problem to include a combination of chemotherapy and immunotherapy treatments with two-contrail variable. Numerical simulations and conclusion are given in Sections 8 and 9.

## 2. Description of the Model

Let us recall Kuznetsov et al.'s model [3] that describes the dynamics of tumour immune system interactions by incorporating a system of five ordinary differential equations (ODEs) model; then we reduce it into two equations but with time delays. The model describes the response of the effector cells (ECs) to the growth of tumour cells (TCs). The penetration of TCs by ECs has been taken into account, which simultaneously causes the inactivation of ECs. It is assumed that interactions between the ECs and TCs are* in vitro* such that *E*(*t*), *T*(*t*), *C*(*t*), E*(*t*), and T*(*t*) denote the local concentrations of ECs, TCs, EC-TC conjugates, inactivated ECs, and “lethally hit” TCs, respectively. The total population of unattacked TCs is *T*_tot_(*t*) = *T*(*t*) + *C*(*t*). The rate of binding of ECs to TCs and the rate of separation of ECs from TCs without damaging them are denoted by *k*_1_ and *k*_−1_, respectively. The rate at which EC-TC integrations program for lysis is denoted by *k*_2_ while the rate at which EC-TC interaction inactivate ECs is denoted by *k*_3_. The model takes the form

(1) dE(t)dt=σ+F(C(t),T(t))−d1E(t)−k1E(t)T(t)+(k−1+k2)C(t),dT(t)dt=αT(t)(1−βTtot(t))−k1E(t)T(t)+(k−1+k3)C(t),dC(t)dt=k1E(t)T(t)−(k−1+k2+k3)C(t),dE∗(t)dt=k3C(t)−d2E∗(t),dT∗(t)dt=k2C(t)−d3T∗(t).

Here, the parameter *σ* represents the normal rate (not increased by the presence of the tumour) of the flow of adult ECs into the tumour side (region), *F*(*C*(*t*), *T*(*t*)) = *F*(*E*(*t*), and *T*(*t*)) > 0 (when *T*(*t*) > 0) describes the accumulation of ECs in the tumour side due to the presence of the tumour. However, *d*_1_, *d*_2_, and *d*_3_ are the coefficients of the processes of destruction and migration of *E*, E*, and T*, respectively. The maximal growth of tumour is represented by the coefficient *α*, and *β* ^−1^ is the environment capacity. If we assume that *dC*(*t*)/*dt* ≈ 0, therefore *C*(*t*) ≈ *KE*(*t*)*T*(*t*) where *K* = *k*_1_/(*k*_−1_ + *k*_2_ + *k*_3_), and the model can be reduced to two equations which describe the behavior of ECs and TCs only [2, 3]. That leads to the fact that the rate of stimulated accumulation has some maximum value as *TCs* get large.

Thus, the reduced model that describes the interactions between the two populations, tumour cells *T*(*t*) and activated effector cells *E*(*t*) (such as cytotoxic T-cells or natural killer cells), is of the form

(2) dE(t)dt=σ+F(E(t),T(t))−μE(t)T(t)−δE(t),dT(t)dt=αT(t)(1−βT(t))−nE(t)T(t),

with the initial conditions *E*(0) = *E*_0_ and *T*(0) = *T*_0_. The interaction between the effector cells and tumour cells can reduce the size of both populations with different rates. This is expressed as −*μE*(*t*)*T*(*t*) and −*nE*(*t*)*T*(*t*) to illustrate the interaction between the tumour cells and effector cells. As a result of this interaction, the immune effector cells decrease the population of tumour cells at the rate *n*. Additionally, tumour cells infect some of the effector cells and, therefore, the population of uninfected effector cells decreases at the rate *μ*.

If one considers *T*(*t*) as prey and *E*(*t*) as predator, then *F*(*E*, *T*) may take the form *F*(*E*, *T*) = *ρE*(*t*)*T*(*t*)/(*η* + *T*(*t*)) which is Michaelis-Menton form. In this term, *ρ* is the maximum immune response rate and *η* is the steepness of immune response. The presence of the tumour cells virtually initiates the proliferation of tumour-specific effector cells to reach a saturation level parallel to the increase in the tumour populations. Hence, the recruitment function should be zero in the absence of the tumour cells, whereas it should increase monotonically towards a horizontal asymptote [23]. We also incorporate a discrete time-delay *τ* into the model, to describe the time needed by the immune system to develop a suitable response after recognizing the tumour cells. Accordingly, the model with discrete time delay takes the form

(3) dE(t)dt=σ+ρE(t−τ)T(t−τ)η+T(t−τ)−μE(t−τ)T(t−τ)−δE(t),dT(t)dt=r2T(t)(1−βT(t))−nE(t)T(t),t≥0,

with the initial functions *E*(*t*) = *ψ*_1_(*t*) and *E*(*t*) = *ψ*_2_(*t*), for all *t* ∈ [−*τ*, 0]. Here *σ* represents the normal rate (not increased by the presence of the tumour) of the flow of adult effector cells into the tumour side (region). The source of the immune cells is considered to be outside of the system, so it is reasonable to assume a constant influx rate *σ*. Furthermore, in the absence of any tumour, the cells will die at the rate *δ*. The presence of tumour cells stimulates the immune response, represented by the positive nonlinear growth term for the immune cells *ρE*(*t* − *τ*)*T*(*t* − *τ*)/(*η* + *T*(*t* − *τ*)) where *ρ* and *η* are positive constants and *τ* ≥ 0 is the time delay that presents the time needed by the immune system to develop a suitable response after recognizing the tumour cells. The saturation term (Michaelis-Menton form) with the *E*(*t*) compartment and logistic term with the *T*(*t*) compartment are consoled. The presence of the tumour cells virtually initiates the proliferation of tumour-specific effector cells to reach a saturation level parallel to the increase in the tumour populations. Let us first prove the nonnegativity and boundedness solutions of the underlying DDEs model (3) (see [24]).

### 2.1. Nonnegativity and Boundedness Solutions of Model (3)

To show that the solutions of model (3) are bounded and remain nonnegative in the domain of its application for sufficiently large values of time *t*, we recall the following lemma.

**Lemma 1 (Gronwall's Lemma [25, page 9]).**

Let *x*, *ψ*, and *χ* be real continuous functions defined in [*a*, *b*], *χ* ≥ 0 for *t* ∈ [*a*, *b*]. One supposes that on [*a*, *b*] one has the inequality *x*(*t*) ≤ *ψ*(*t*) + ∫_*a*_^*t*^ *χ*(*s*)*x*(*s*)*ds*. Then *x*(*t*) ≤ *ψ*(*t*) + ∫_*a*_^*T*^ *χ*(*s*)*ψ*(*s*)*e* ^(∫_*s*_^*t*^*χ*(*ξ*)*dξ*)^ *ds* in [*a*, *b*].

Therefore, we arrive at the following proposition.

**Proposition 2 .**

Let (*E*, *T*) be a solution of the DDEs model (3); then *E*(*t*) < *M*_1_ and *T*(*t*) < *M*_2_ for all sufficiently large time *t*, where

(4) M1=E(0)+σδexp⁡(δt)+∫0t[ρeδ(τ+s)(E(0)+σδeδs)×exp⁡(∫stρeδ(τ+ξ)dξ)]ds,M2=max⁡(1β,T(0)).

**Proof**

Let (*E*, *T*) denote the solution of model (3). From the second equation of system (3), we have *dT*/*dt* ≤ *r*_2_ *T*(*t*)(1 − *βT*(*t*)). Thus, *T*(*t*) may be compared with the solution of

(5) $$\begin{array}{c} \frac{dX}{dt}=r_{2}X\left(t\right)\left(1-\beta X\left(t\right)\right),\;\text{with}\;\;X\left(0\right)=T\left(0\right). \end{array}$$

This proves that *T*(*t*) < *M*_2_. From the first equation of system (3), we obtain

(6) E(t)=exp⁡(−δt) ×{E(0)+∫0t[σ+ρE(s−τ)T(s−τ)η+T(s−τ)−μE(s−τ)T(s−τ)]×exp⁡(δs)ds}.

To show that *E*(*t*) is bounded, we use the generalized Gronwall Lemma. Since *T*/(*η* + *T*) < 1 and exp⁡ (−*δt*)∈(0,1], we have

(7) $$\begin{array}{c} E\left(t\right)\leq E_{0}+\frac{\sigma }{\delta }\exp\left(\delta t\right)+\overset{t}{\underset{0}{\int }}\rho E\left(s-\tau \right)\exp\left(\delta s\right)ds. \end{array}$$

The generalized Gronwall Lemma gives *E*(*t*) < *M*_1_ where *M*_1_ is uniformly bounded. It follows that if (*E*, *T*) is a solution of (3), then (*E*, *T*)<(*M*_1_, *M*_2_) for all *t*. This shows that the solutions of model (3) are uniformly bounded. This completes the proof.

From (1) and the solution *T*(*t*) = *T*(0)exp⁡ (∫_0_^*t*^[*r*_2_(1 − *βT*(*s*)) − *E*(*s*)]*ds*), we arrive at the following result.

**Corollary 3 .**

If *ρ*/(*η* + *T*) ≥ *μ*, then the solutions (*E*, *T*) for model (3) are nonnegative for any nonnegative initial condition. However, if *ρ*/(*η* + *T*) < *μ*, then there exist nonnegative initial conditions such that *E*(*t*) becomes negative in a finite time interval.

### 2.2. Model with Chemotherapy

**Figure 1:**
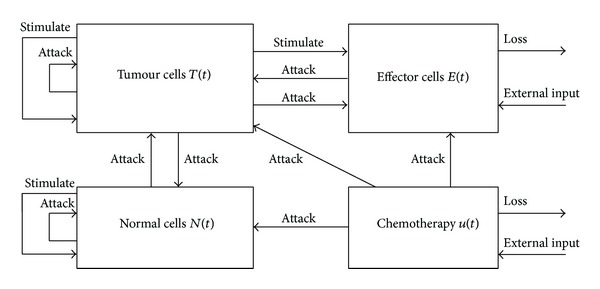
The interaction of tumour cells, immune cells, and normal cells in the presence of chemotherapy drug.

We extend model (3) to consider extra two variables, namely, amount of chemotherapy, *u*(*t*), and normal cells, *N*(*t*) (see Figure 1). We also assume a homogeneity of the tumour cells. The modified model is

(8) dE(t)dt=σ+ρE(t−τ)T(t−τ)η+T(t−τ)−μE(t−τ)T(t−τ)−δE(t)−a1(1−e−u(t))E(t),dT(t)dt=r2T(t)(1−βT(t))−nE(t)T(t)−c1N(t)T(t)−a2(1−e−u)T(t),dN(t)dt=r3N(t)(1−β2N(t))−c2T(t)N(t)−a3(1−e−u)N(t),du(t)dt=v(t)−d1u(t).

We assume that the drug kills all types of cells but that the killing rate differs for each type of cells; *F*(*u*) = *a*_*i*_(1 − *e* ^−*u*^) is the fraction cell kill for a given amount of drug, *u*(*t*), at the tumour site. We denote by *a*_1_, *a*_2_, and *a*_3_ the three different response coefficients. *v*(*t*) represents the amount of dose that is injected into the system, while *d*_1_ is the decay rate of the drug once it is injected. In this case, the quantity we will control directly is not *u*(*t*) but *v*(*t*). The tumour cells and normal cells are modelled by a logistic growth law, with the parameters *r*_*i*_ representing the growth rate of two types of cells: *i* = 2 identifies the parameter associated with tumour, and *i* = 3 identifies the one associated with the normal tissue; *β* and *β*_2_ are the reciprocal carrying capacities of tumour cells and host cells, respectively. In addition, there are two terms representing the competition between the tumour and host cells −*c*_1_ *NT* and −*c*_2_ *NT*.

Let *C* = *C*([−*τ*, 0], *R* ^4^) be the Banach space of continuous functions mapping the interval [−*τ*, 0] into *R* ^4^ with the topology of uniform convergence. It is easy to show that there exists a unique solution (*E*(*t*), *T*(*t*), *N*(*t*), *u*(*t*)) of system (8) with initial data (*E*_0_, *T*_0_, *N*_0_, *u*_0_) ∈ *C*. For biological reasons, we assume that the initial data of system (8) satisfy *E*_0_ ≥ 0, *T*_0_ ≥ 0, *N*_0_ ≥ 0, and *u*_0_ ≥ 0. For *τ* = 0, the model is reduced to ODEs model developed by De Pillis and Radunskaya in [26].

**Remark 4 .**

We consider that the units of the model cells are normalized, so that *β*_2_ = 1.

The main objective in developing chemotherapy treatment, in system (8), is to reach either tumour-free steady state or coexisting steady state in which the tumour cells' size is small, while the normal cells' size is closed to its normalized carrying capacity. We next start the analysis with studying the stability of the system when there is no drug (treatment) input; that is, *u*(*t*) = 0, for all *t*.

## 3. Drug-Free Steady States and Their Stability

In the absence of chemotherapy (*u*(*t*) = 0), model (8) has the following types of steady states:

(a) tumour-free steady state, where the tumour cells population is zero, while the normal cells survive. This steady state has the form *E*_0_ = (*σ*/*δ*, 0,1);
(b) dead (lethal) steady state, where the normal cells population is zero, which has the following forms:
  (i) (*σ*/*δ*, 0,0) in which the normal and tumour cell populations have died off,
  (ii) (*f*(T*), T*, 0) where the normal cells alone have died off and the tumour cells have survived, where
    (9) $$\begin{array}{c} f\left(T\right)=\frac{\sigma \left(\eta +T\right)}{\mu T\left(\eta +T\right)+\delta \left(\eta +T\right)-\rho T}, \end{array}$$
    and T* is a nonnegative solution for
    (10) $$\begin{array}{c} T^{*}+\left(\frac{n}{r_{2}\beta }\right)f\left(T^{*}\right)-\frac{1}{\beta }=0; \end{array}$$
(c) coexisting steady state, where both normal and tumour cells coexist with nonzero populations. The steady state is given by *E*_+_ = (*f*(T*), T*, *g*(T*)) where *g*(T*) = 1 − (*c*_2_/*r*_3_)T*, and T* is a nonnegative solution of
  (11) C3T3+C2T2+C1T+C0=0, whereC3=−μr2β+μc1c2r3,C2=−μηr2β+μηc1c2r3+μr2−μc1−δr2β   +δc1c2r3+ρr2β−ρc1c2r3,C1=μηr2−μηc1−δηr2β+δηc1c2r3+δr2   −δc1−ρr2+ρc1−σn,C0=δηr2−δηc1−σnη.

**Figure 2:**
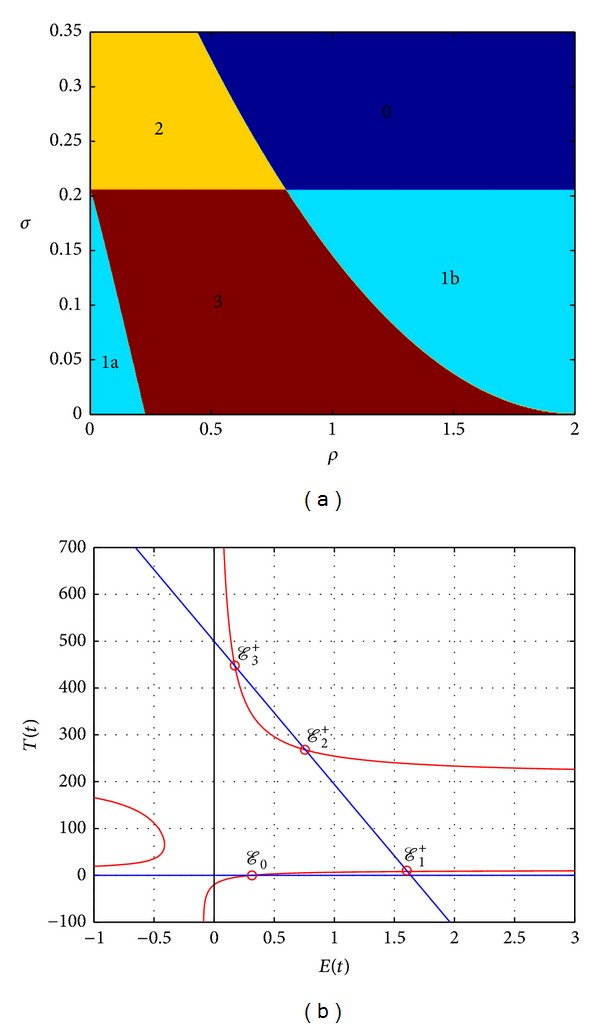
(a) shows the regions of existence of coexisting equilibria of model (3) in (*ρ*, *σ*)-plane with the parameter values *δ* = 0.2, *η* = 0.3, *μ* = 0.003611, *r*_2_ = 1.03, *r*_3_ = 1, *β* = 2 × 10^−3^, *n* = 1, *c*_1_ = 0.00003, and *c*_2_ = 3 × 10^−9^. The dark blue region (0) represents the case where there is no equilibrium, the light blue regions (1a, 1b) represent the case where there is a unique equilibrium, the orange region (2) represents the case where there are two steady states, and the brown region (3) represents the case where there is three equilibria. (b) shows the nullclines of the model which has up to four steady states: tumour-free steady state “*E*_0_”; tumour dormancy steady state “*E*_1_^+^”; medium concentration tumour steady state “*E*_2_^+^”; and escape tumour steady state “*E*_3_^+^.”

The number of coexisting steady states mainly depends on the parameter values. There could be zero, one, two, or three of these steady states (see Figure 2). We next study the stability of the previously mentioned steady states.

### 3.1. Stability of Tumour-Free Steady State

In this subsection, we investigate the parameter ranges for which the tumour-free steady state *E*_0_ is locally asymptotically stable. The Jacobian matrix of the linearized system at the tumour-free steady state is given by

(12) $$\begin{array}{c} J_{E_{0}}=\left(\begin{array}{ccc} -\delta & \frac{\rho \sigma }{\eta \delta }e^{-\lambda \tau }-\frac{\mu \sigma }{\delta }e^{-\lambda \tau } & 0\\ 0 & r_{2}-\frac{n\sigma }{\delta }-c_{1} & 0\\ 0 & -c_{2} & -r_{3} \end{array}\right) \end{array}$$

with the eigenvalues *λ*_1_ = −*δ* < 0, *λ*_2_ = *r*_2_ − *nσ*/*δ* − *c*_1_, and *λ*_3_ = −*r*_3_ < 0. Hence, the tumour-free steady state *E*_0_ is locally stable if *λ*_2_ < 0 if and only if

(13) $$\begin{array}{c} r_{2}<\frac{n\sigma }{\delta }+c_{1},\;\forall \tau \geq 0. \end{array}$$

This relates *r*_2_ (the growth rate of the tumour cells) to the *nσ*/*δ* (the resistance coefficient), which measures how efficiently the immune system competes with the tumour cells. If this tumour-free steady state is unstable, then no amount of chemotherapy will be able to completely eradicate the tumour cells.

### 3.2. Stability of Lethal Steady States

The same analysis done above shows that the deadly steady state (*σ*/*δ*, 0,0) has the eigenvalues *λ*_1_ = −*δ* < 0, *λ*_2_ = *r*_2_ − *nσ*/*δ*, and *λ*_3_ = *r*_3_ > 0 and hence it is unstable saddle point, while the other deadly steady state (*f*(T*), T*, 0) can be either stable or unstable depending on the model parameters and the value of the time-delay *τ*. This can be shown by using Routh Harwatz test and Rouche's theorem as shown in detail in the previous chapters. Since the stability of this steady state is not needed for the developing treatment therapy, we will not introduce more details in this part.

### 3.3. Stability of Coexisting Steady States

**Figure 3:**
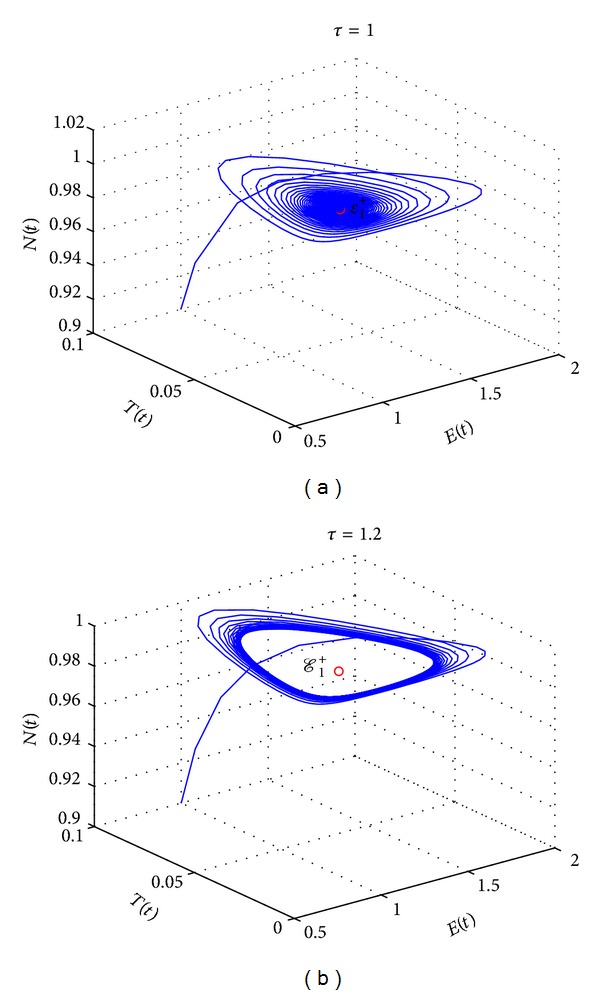
The phase space for the cell populations in the case where *ρ* = 1.4 and *σ* = 0.1. (a) shows that, for *τ* = 1, the steady state is asymptotically stable. (b) shows that, for *τ* = 1.2, a limit cycle is born around the steady state.

To study the stability of the coexisting steady states, we vary the two parameters *ρ* (the immune cells growth rate) and *σ* (the normal flow rate of immune cells), with fixing the other parameters: *δ* = 0.2, *η* = 0.3, *μ* = 0.003611, *r*_2_ = 1.03, *r*_3_ = 1, *β* = 2 × 10^−3^, *n* = 1, *c*_1_ = 0.00003, and *c*_2_ = 3 × 10^−9^.  Table 1 summarizes the existence and stability results of the coexisting steady states as present in different regions of Figure 2. It shows that the light blue region (1a) represents the “escape” case where there is a unique stable node steady state with high tumour size, while the light blue region (1b) represents the case where there is a unique steady state with low tumour size. It is stable spiral for *τ* < τ*, while at *τ* = τ* the limit cycle occurs due to Hopf bifurcation. Furthermore, the orange region (2) represents the case where there are two steady states; one is stable node and the other is unstable sacdle node. To this end, the brown region (3) represents the case where there are three steady states: one is stable node, one is unstable node, and the last steady state is spiral stable for *τ* < τ*, while the limit cycle occurs at *τ* = τ*. Of interest are the existence and stability of steady states where a small tumour population size might coexist with a large number of normal cells.  Figure 3 presents the phase space for the cell populations in the case where *ρ* = 1.4 and *σ* = 0.1. It shows that, for *τ* = 0.8, the steady state is asymptotically stable (Figure 3(a)), while, for *τ* = 1.2, a limit cycle is born around the steady state (Figure 3(b)). We utilize MIDDE code [27] to solve the DDEs model, which is suitable to simulate stiff and nonstiff problems, using monoimplicit RK methods [28]. We next consider the chemotherapy treatment (*u*(*t*) > 0) in the underlying model and establish the existence of an optimal control for the model and provide necessary conditions for the optimal control.

## 4. Optimal Control Problem Governed by DDEs

Once a suitable model of interacting cell populations is constructed, we then focus on the design of an efficient treatment protocol, where we employ the tools of optimal control theory.

Consider the optimal control problem with pure state constraints and control bounds, as follows:

(14a) $$\begin{array}{c} \underset{x,v}{\max}\;J\left(x,v\right)=\Psi \left(x\left(t_{f}\right)\right)+\overset{t_{f}}{\underset{0}{\int }}L\left(t,x\left(t\right),v\left(t\right)\right)dt, \end{array}$$

subject to the DDEs

(14b) $$\begin{array}{c} x^{\prime }\left(t\right)=f\left(t,x\left(t\right),x\left(t-\tau \right),v\left(t\right)\right),\;t\in \left[0,t_{f}\right], \end{array}$$

(14c) $$\begin{array}{c} x\left(t\right)=\phi \left(t\right),\;t\in \left[-\tau ,0\right], \end{array}$$

with the control constraint

(14d) $$\begin{array}{c} a\leq v\left(t\right)\leq b,\;t\in \left[0,t_{f}\right] \end{array}$$

and state constraint

(14e) $$\begin{array}{c} x\left(t\right)\geq c,\;t\in \left[0,t_{f}\right]. \end{array}$$

*J* is called objective functional and the integrand *L*(·) is called the Lagrangian of objective functional. Furthermore, *a* and *b* are called the lower and upper bounds. The function *v*(*t*) is called an admissible control if and only if it fulfills inequality constraints (14d). The set of all admissible controls is called the admissible set and we referred to it by *V*_ad_ (where “ad” stands for the admissible). The state *x*(·) enters with a delay *τ* as *x*(*t* − *τ*) in the system of the state equations (14b) while it is evaluated at the time *t* as *x*(*t*) in the objective functional (14a). The set of all admissible states *X*_ad_, which satisfy the state equations and the state constraint, is called the set of admissible state.

The goal of chemotherapy is to eradicate the tumour cells, while maintaining adequate amounts of healthy tissue. From a mathematical point of view, adequate destruction to tumour cells might mean forcing the system out of the basin of an unhealthy spiral node, out of a limit cycle, and into a basin of attraction of a stable tumour-free equilibrium. Alternatively, if the therapy pushes the system into a limit cycle in which the size of the tumour is small for a long period of time (as long as the life of the patient, for example), this could also be considered a “cure.”

Optimality in treatment might be defined in a variety of ways. Some studies have been done in which the total amount of drug administered is minimized, or the number of tumour cells is minimized. The general goal is to keep the patient healthy while killing the tumour. Since our model takes into account the toxicity of the drug to all types of cells, our control problem consists of determining the function *v*(*t*) that will maximize the amount of effector cells and minimize the number of tumour cells and the cost of the control with the constraint that we do not kill too many normal cells. If the units of cells are normalized, so that the carrying capacity of normal cells is 1, we then require that the number of normal cells stays above three-fourths of the carrying capacity. Therefore, our main objective is to optimize the functional

(15a) $$\begin{array}{c} \underset{v\in V_{\text{ad}}}{\max}\;J\left(v\right)=\overset{t_{f}}{\underset{0}{\int }}\left(E\left(t\right)-T\left(t\right)-\frac{B_{v}}{2}{\left[v\left(t\right)\right]}^{2}\right)dt \end{array}$$

which subject to the underlying DDEs

(15b) dE(t)dt=σ+ρE(t−τ)T(t−τ)η+T(t−τ)     −μE(t−τ)T(t−τ)−δE(t)     −a1(1−e−u(t))E(t),

(15c) dT(t)dt=r2T(t)(1−βT(t))−nE(t)T(t)     −c1N(t)T(t)−a2(1−e−u)T(t),

(15d) dN(t)dt=r3N(t)(1−β2N(t))−c2T(t)N(t)     −a3(1−e−u)N(t),

(15e)  du(t)dt=v(t)−d1u(t)

with control constraint

(15f) $$\begin{array}{c} 0\leq v\left(t\right)\leq v_{\max}<\infty ,\;t\in \left[0,t_{f}\right] \end{array}$$

and state constraint

(15g) $$\begin{array}{c} k\left(N\right)=N-0.75\geq 0,\;t\in \left[0,t_{f}\right]. \end{array}$$

Here, *B*_*v*_ is a weight factor that describes the patient's acceptance level of chemotherapy. We choose the control parameter as a class of piecewise continuous functions defined for all *t* such that 0 ≤ *v*(*t*) ≤ *v*_max⁡_ < *∞*, where *v*(*t*) = *v*_max⁡_ represents the maximum chemotherapy, while *v*(*t*) = 0 represents the case where there is no chemotherapy. Thus, we depict the class of admissible controls as

(16) Vad={v∈L∞([0,tf],R) ∣ 0≤v(t)≤vmax⁡<∞,∀t∈[0,tf]}.

We next prove the existence of the optimal solution of the underlying system (15a)–(15g).

## 5. Existence of an Optimal Solution

To prove the existence of the optimal solution of (15a)–(15g), we use the results of Fleming and Rishel [29, Theorem 4.1, pages 68-69] and Lukes [30, Theorem 9.2.1, page 182].

**Theorem 5 .**

There exists an optimal solution (x*, v*) ∈ *W* ^1,*∞*^([0, *t*_*f*_], *R* ^4^) × *L* ^*∞*^([0, *t*_*f*_], *R*) for the optimal control problem (15a)–(15g) such that

(17) $$\begin{array}{c} J\left(v^{*}\right)=\underset{v\in V_{ad}}{\max}\;J\left(v\right), \end{array}$$

where x* = [E*, T*, N*, u*]^*T*^ if the following conditions are satisfied.

1. The set of admissible state is nonempty.
2. The admissible set *V*_*ad*_ is nonempty, convex, and closed.
3. The right-hand side of the state system is bounded by a linear combination of the state and control variables.
4. The integrand, *L*(*E*, *T*, *v*) = (*E*(*t*) − *T*(*t*) − (*B*_*v*_/2)[*v*(*t*)]^2^), of the objective functional is a concave on *V*_*ad*_.
5. There exist constants *h*_2_, *h*_3_ > 0, and *b* > 1 such that *L*(*E*, *T*, *v*) ≤ *h*_2_ − *h*_3_(|*v*|)^*b*^.

**Proof**

In order to verify the above conditions, we should first prove the existence of the solution for system of the state equations (15b)–(15e). Since *ρT*(*t* − *τ*)/(*η* + *T*(*t* − *τ*)) < *ρ*, *v*_max⁡_ < *∞* and, by neglecting the negative terms in the model, we have

(18) $$\begin{array}{c} \frac{dE\left(t\right)}{dt}<\sigma +\rho E\left(t-\tau \right),\;\;\frac{dT\left(t\right)}{dt}<r_{2}T,\\ \frac{dN\left(t\right)}{dt}<r_{3}N,\;\;\frac{du\left(t\right)}{dt}<v_{\max}. \end{array}$$

System (18) can be rewritten in the form

(19) (E(t)T(t)N(t)u(t))′<(00000r20000r300000)(E(t)T(t)N(t)u(t)) +(ρ000000000000000)(E(t−τ)T(t−τ)N(t−τ)u(t−τ))+(σ00vmax⁡),

where ′ = *d*/*dt*. This system is linear in finite time with bounded coefficients. Then the solutions of this linear system are uniformly bounded. Therefore, the solution of the nonlinear system (15b)–(15e) is bounded and exists [30]. Hence, condition one is satisfied.

Clearly, the second condition is satisfied by the definition of *V*_ad_. System (15b)–(15e) is bilinear in the control variable *v* and can be rewritten as

(20) $$\begin{array}{c} \vec{F}\left(t,\vec{X}\left(t\right),\vec{X}\left(t-\tau \right),v\right)=\vec{\alpha }\left(t,\vec{X}\right)+\vec{\beta }\left(t,\vec{X}\left(t-\tau \right)\right)+\sigma +v, \end{array}$$

where $\vec{X}(t)=(E,T,N,u)$, $\vec{X}\left(t-\tau \right)=(E(t-\tau ),\;T(t-\tau ),\;N(t-\tau ),\;u(t-\tau ))$, and $\vec{\alpha }$ and $\vec{\beta }$ are the vector valued functions of $\vec{X}(t)$ and $\vec{X}(t-\tau )$, respectively. Using the fact that the solutions are bounded, we have

(21) |F→(t,X→(t),X→(t−τ),v)| ≤|F1X(t)|+|F2X(t−τ)|+|F3|+|F4| ≤h1|X→|+|σ|+|v|,

where *h*_1_ depends on the coefficients of the system, and

(22) $$\begin{array}{c} F_{1}=\left(\begin{array}{cccc} 0 & 0 & 0 & 0\\ 0 & r_{2} & 0 & 0\\ 0 & 0 & r_{3} & 0\\ 0 & 0 & 0 & 0 \end{array}\right),\;\;F_{2}=\left(\begin{array}{cccc} \rho & 0 & 0 & 0\\ 0 & 0 & 0 & 0\\ 0 & 0 & 0 & 0\\ 0 & 0 & 0 & 0 \end{array}\right),\\ F_{3}=\left(\begin{array}{c} \sigma \\ 0\\ 0\\ 0 \end{array}\right),\;\;F_{4}=\left(\begin{array}{c} 0\\ 0\\ 0\\ v \end{array}\right). \end{array}$$

We also note that the integrand of *J*(*v*) is concave in *V*_ad_. Finally,

(23) E(t)−T(t)−Bv2[v(t)]2<E−Bv2[v(t)]2≤h2−h3|v(t)|2,

where *h*_2_ depends on the upper bounds of *E*(*t*) and *T*(*t*), and *h*_3_ = *B*_*v*_/2. This completes the proof.

We also conclude that there exists an optimal control variable v*.

## 6. Optimality Conditions

In this section, we establish the necessary conditions for the optimal solution of the optimization problem (15a)–(15g); we use Pontryagin's minimum (maximum) principle derived by Göllmann et al. [31] for the retarded optimal control problem with mixed control-state constraints. To this end, we define the augmented Hamiltonian function involving the inequality constraints by

(24) H(t,E,T,Eτ,Tτ,u,v,λ) =E(t)−T(t)−Bv2[v(t)]2+λ1(T)dE(t)dt  +λ2(t)dT(t)dt+λ3(t)dN(t)dt+λ4du(t)dt  +γ(t)k(N),

where

(25) $$\begin{array}{c} \gamma \left(t\right)=\{\begin{array}{cc} 1 & \text{if}\;N\left(t\right)\leq 0.75,\\ 0 & \text{otherwise} \end{array} \end{array}$$

and *λ*_*i*_ (*i* = 1,2, 3,4) are the adjoint variables that satisfy

(26) λ1′(t)=−∂H∂E(t)−χ[0,tf−τ](t)∂H∂Eτ(t+τ), λ1(tf)=0,λ2′(t)=−∂H∂T(t)−χ[0,tf−τ](t)∂H∂Tτ(t+τ), λ2(tf)=0,λ3′(t)=−∂H∂N(t),  λ3(tf)=0,λ4′(t)=−∂H∂u(t),  λ4(tf)=0.

Here *χ*_[0,*t*_*f*_−*τ*]_ denotes the indicator function of the interval [0, *t*_*f*_ − *τ*] and defined by

(27) $$\begin{array}{c} \chi_{\left[0,t_{f}-\tau \right]}=\{\begin{array}{cc} 1 & \text{if}\;t\in \left[0,t_{f}-\tau \right],\\ 0 & \text{otherwise}. \end{array} \end{array}$$

To minimize the Hamiltonian functional, the Pontryagin's minimum principle [31] is used. Thus, we arrive at the the following theorem.

**Theorem 6 .**

Let (x*, v*) ∈ *W* ^1,*∞*^([0, *t*_*f*_], *R* ^4^) × *L* ^*∞*^([0, *t*_*f*_], *R*) be the optimal solutions of (15a)–(15g), where x* = [E*, T*, N*, u*]^*T*^. Then, there exists an adjoint state *λ*(*t*) ∈ *W* ^1,*∞*^([0, *t*_*f*_], *R* ^4^), defined by (26), such that the triple (x*, v*, *λ*) satisfies the state equation

(28) dE∗(t)dt=σ+ρE∗(t−τ)T∗(t−τ)η+T∗(t−τ)−μE∗(t−τ)T∗(t−τ)−δE∗(t)−a1(1−e−u∗)E∗(t),dT∗(t)dt=r2T∗(t)(1−βT∗(t))−μE∗(t)T∗(t)−c1N∗(t)T∗(t)−a2(1−e−u∗(t))T∗(t),dN∗(t)dt=r3N∗(t)(1−β2N∗(t))−c2T∗(t)N∗(t)−a3(1−e−u∗(t))N∗(t),du∗(t)dt=v∗(t)−d1u∗(t),

with the initial conditions

(29) E∗(t)=ϕ1(t),  T∗(t)=ϕ2(t),N∗(t)=ϕ3(t),  u(t)=ϕ4(t),t∈[−τ,0],

the adjoint state equations

(30) λ1′(t)=−1+λ1(t)[δ+a1(1−e−u∗)] +λ2(t)nT∗+λ1(t+τ)χ[0,tf−τ][μT∗−ρT∗η+T∗],λ2′(t)=1+λ2[−r2+2r2βT∗+nE∗+c1N∗+a2(1−e−u∗)]+λ3c2N∗ +χ[0,tf−τ]λ1(t+τ)[ρE∗T∗(η+T∗)2−ρE∗η+T∗+μE∗],λ3′(t)=λ2c1T∗−λ3(r3−2r3β2N∗−c2T∗−a3(1−e−u∗))−γ,λ4′(t)=−λ1(t)a1e−u∗E∗+λ2(t)a2e−u∗T∗ +λ3(t)a3e−u∗N∗+λ4(t)d1,

with transversality conditions

(31) $$\begin{array}{c} \lambda_{i}\left(t_{f}\right)=0,\;i=1,2,3,4, \end{array}$$

and the optimal control

(32) $$\begin{array}{c} v^{*}=\min\left(v_{\max},\frac{\lambda_{4}}{B_{v}}\right). \end{array}$$

**Proof**

The optimal control v* can be solved from the optimality condition (∂*H*/∂*v*)(*t*) = 0; that is, −*B*_*v*_ *v* + *λ*_4_ = 0. By using the handedness of the control set *V*_ad_, it is easy to obtain v* in the form of (32).

## 7. Immunochemotherapy

Model (8) is extended to include external source of immunotherapy treatment of the effector cells such as ACI. We then add the term *w*(*t*)*s*_1_ to represent the input rate of externally administered antitumour effector cells, where *w*(*t*) is the control parameter. Our goal is to maximize an objective functional *J* subject to the new model with a combination of chemotherapy and ACI and constraints on the control and the state:

(33a) max⁡v,w∈Wad J(v,w)=∫0tf(E(t)−T(t)−[Bv2[v(t)]2+Bw2[w(t)]2])dt,

subject to DDEs

(33b) dE(t)dt=σ+ρE(t−τ)T(t−τ)η+T(t−τ)−μE(t−τ)T(t−τ)     −δE(t)−a1(1−e−u(t))E(t)+w(t)s1,

(33c) dT(t)dt=r2T(t)(1−βT(t))−nE(t)T(t)−c1N(t)T(t)     −a2(1−e−u(t))T(t),

(33d) dN(t)dt=r3N(t)(1−β2N(t))−c2T(t)N(t)     −a3(1−e−u(t))N(t),

(33e)  du(t)dt=v(t)−d1u(t),

the control constraints

(33f) 0≤v(t)≤vmax⁡<∞,  0≤w(t)≤wmax⁡<∞,t∈[0,tf],

and the state constraint

(33g) $$\begin{array}{c} k\left(N\right)=N-0.75\geq 0,\;t\in \left[0,t_{f}\right], \end{array}$$

where *B*_*w*_ is a weight factor that describes a patient's acceptance level of immunotherapy and the set of all admissible controls *W*_ad_ is defined by

(34) Wad={(v,w):(v,w)  piecewise continuous, such that0≤v(t)≤vmax⁡<∞,  0≤w(t)≤wmax⁡<∞,∀t∈[0,tf]}.

Similarly, the optimal solution of the optimization problem (33a)–(33g) satisfies the state equations

(35) dE∗(t)dt=σ+ρE∗(t−τ)T∗(t−τ)η+T∗(t−τ)−μE∗(t−τ)T∗(t−τ)−δE∗(t)−a1(1−e−u∗)E∗(t)+w∗(t)s1,dT∗(t)dt=r2T∗(t)(1−βT∗(t))−μE∗(t)T∗(t)−c1N∗(t)T∗(t)−a2(1−e−u∗(t))T∗(t),dN∗(t)dt=r3N∗(t)(1−β2N∗(t))−c2T∗(t)N∗(t)−a3(1−e−u∗(t))N∗(t),du∗(t)dt=v∗(t)−d1u∗(t),E∗(t)=ϕ1(t),  T∗(t)=ϕ2(t),N∗(t)=ϕ3(t),  u(t)=ϕ4(t),          t∈[−τ,0].

The adjoint state equations are

(36) λ1′(t)=−1+λ1(t)[δ+a1(1−e−u∗(t))] +λ2(t)nT∗(t)+λ1(t+τ)χ[0,tf−τ] ×[μT∗(t)−ρT∗(t)η+T∗(t)],λ2′(t)=1+λ2[−r2+2r2βT∗(t)+nE∗(t)+c1N∗(t)+a2(1−e−u∗)] +λ3c2N∗(t)+χ[0,tf−τ]λ1(t+τ) ×[ρE∗(t)T∗(t)(η+T∗(t))2−ρE∗(t)η+T∗(t)+μE∗(t)],λ3′(t)=λ2c1T∗(t)−λ3(t) ×(r3−2r3β2N∗(t)−c2T∗(t)−a3(1−e−u∗(t))) −γ,λ4′(t)=−λ1(t)a1e−u∗(t)E∗(t)+λ2(t)a2e−u∗(t)T∗(t) +λ3(t)a3e−u∗(t)N∗(t)+λ4(t)d1,

with the transversality conditions *λ*_*i*_(*t*_*f*_) = 0, *i* = {1,2, 3,4}, and the minimum condition

(37) $$\begin{array}{c} v^{*}=\min\left(v_{\max},\frac{\lambda_{4}}{B_{v}}\right),\;\;w^{*}=\min\left(w_{\max},\frac{\lambda_{1}s_{1}}{B_{w}}\right). \end{array}$$

When *s*_1_ = 0 (without immunotherapy), system (35)–(37) reduces to system (28)–(32).

**Remark 7 .**

In the case of immunotherapy alone (*u*(*t*) = 0), the objective functional becomes

(38) $$\begin{array}{c} J\left(w\right)=\overset{t_{f}}{\underset{0}{\int }}\left(E\left(t\right)-T\left(t\right)-\frac{B_{w}}{2}{\left[w\left(t\right)\right]}^{2}\right)dt. \end{array}$$

## 8. Numerical Simulations of the Optimal Control System

Numerical simulations leading to the approximation of the optimal controls (35)–(37) are carried out using the forward Euler method for the state system and backward difference approximation for the adjoint system. We assume the step-size *h*, such that *τ* = *mh* and *t*_*f*_ − *t*_0_ = *nh*, where (*m*, *b*) ∈ *N* ^2^. We define the state, adjoint, and control variables at the mesh points. An initial guess is given for the controls *v* and *w*, which are then updated continuously until the objective functional satisfies the conditions. However, there are several major problems to be overcome when solving delay differential equations. These include stability, stiffness, and discontinuities in the right-hand side of the equation. Stability and stiffness can be handled by the correct choice of implicit solvers [27]. The delay terms can create a whole suite of discontinuities; see [32, 33].

We choose a different set of parameter values (in stable and unstable regions). In the current simulations, we vary the three parameters *σ*, *ρ*, and *τ*, and fix the other parameters:

(39) δ=0.2, η=0.3, μ=0.003611, s1=0.3,r2=1.03, r3=1, β=2×10−3, β2=1,n=1, c1=0.00003, c2=0.00000003, a1=0.2,a2=0.4, a3=0.1, d1=0.01, B=100.

We solve the optimality system to determine the optimal control situation (i.e., the drug strategy) and predict the evolution of the tumour cells, effector cells, and normal cells of each control strategy in 30 days.

**Figure 4:**
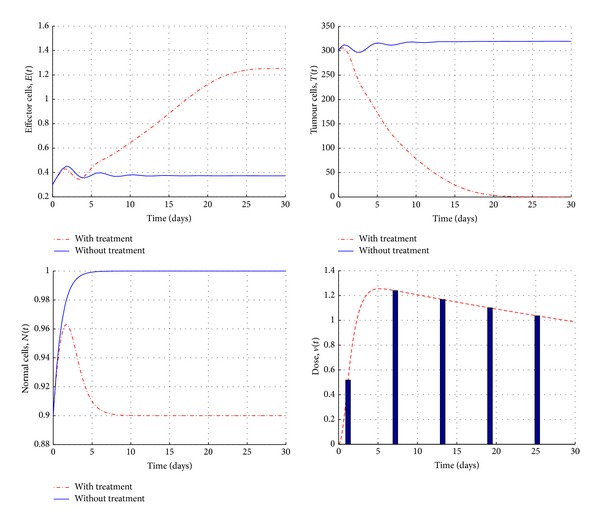
Simulations of system (28)–(32), in the stable region, before and after the treatments with control with the initial conditions *E*_0_ = 0.3, *T*_0_ = 300, and *N*_0_ = 0.9 and the parameter values are given in the text.

**Figure 5:**
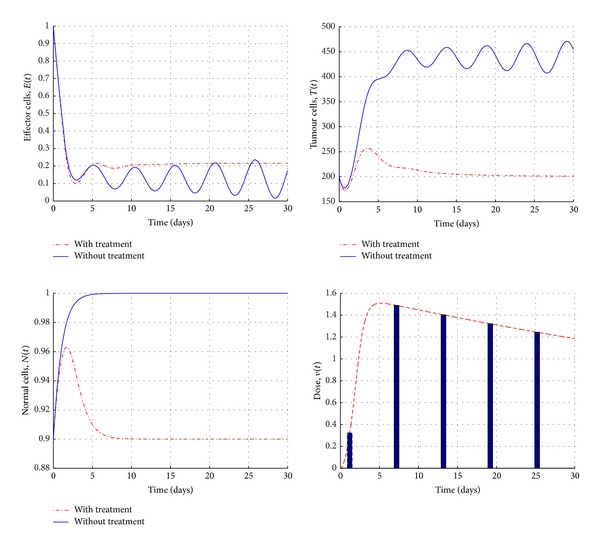
Simulations of the system (28)–(32), in an unstable region, before and after the chemotherapy treatment with the control and initial conditions *E*_0_ = 1, *T*_0_ = 200, and *N*_0_ = 0.9 and the parameter values are given in the text.

Figure 4 shows the numerical simulations of the state system before and after chemotherapy treatment using optimality system (28)–(32) when *σ* = 0.5, *ρ* = 0.01, and *τ* = 1.2 (in the stable region). We note that, in the presence of chemotherapy with optimal control, the effector cells population grows up significantly, while the tumour cells population decreases and is totally eradicated after 20 days. In the meantime, the normal cells population remains over 75%. Yet, Figure 5 shows the impact of chemotherapy treatments (with optimal control) when we choose the parameter values in an unstable region (*σ* = 0.2, *ρ* = 0.2, and *τ* = 1.5). The tumour and effector cells populations are oscillating over time in the absence of chemotherapy, while the presence of treatment helps the immune system to keep the growth of the tumour cells under its control.

**Figure 6:**
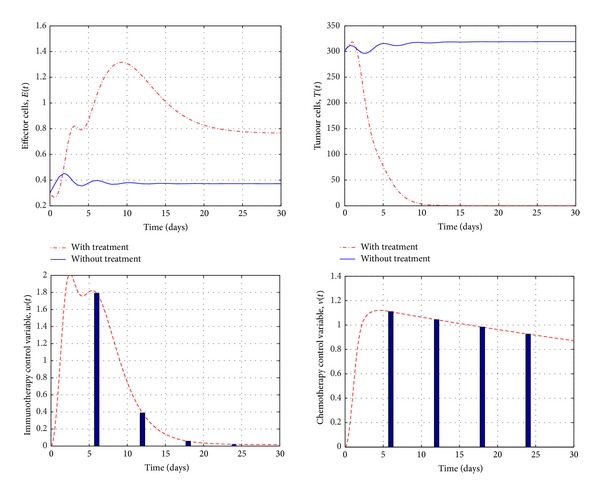
Simulations of system (35)–(37), in the stable region, before and after the immunochemotherapy treatments with controls. It shows that the tumour cells population can be eradicated in day 12.

Figure 6 presents the evolution of system (35)–(37) in the case of combination of chemotherapy and ACI. The parameters values are chosen in the stable region. We notice that the tumour cells population can be eradicated after day 12 which is faster compared to the results of Figure 4 when we used the chemotherapy alone. In other words, the numerical results show that using the combination immunochemotherapy is more effective than using chemotherapy treatment alone.

**Figure 7:**
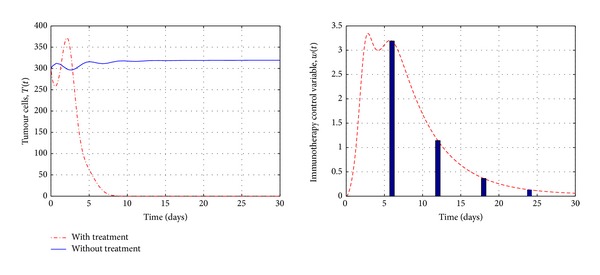
Simulations of the tumour cells population of system (35)–(37), before and after immunotherapy with control. It shows that the tumour cells can be eradicated at day 7 with high immunotherapy dosage where the control value *w*(*t*) reaches the value of 3.5.

However, Figure 7 shows evolution of the system with only immunotherapy (i.e., without chemotherapy). We may notice from the figure that this case reflects the best therapeutic strategies for treatment of tumour, where the recovery becomes faster with high dosage of immunotherapy where *w*(*t*) can reach the value of 3.5 level compared with the combination it was in level 2.

## 9. Concluding Remarks

In this paper, we provided a delay differential model with control variables that describe the interactions of immune cells, tumour cells, normal cells, and immunochemotherapy treatment with control variables. A pontryagin-type maximum principle is derived, for retarded optimal control problems with delays in the state variable when the control system is subject to a mixed controlstate constraint, in order to minimize the cost of treatment, reduce the tumour cells load, and keep the number of normal cells above 75% of its carrying capacity. We presented an efficient numerical technique, based on forward difference approximation to the state system and backward difference scheme to the adjoint system, to solve the optimal control problem and identify the best treatment strategy when we adopt the chemotherapy treatment alone or a combination of chemoimmunotherapy, with minimum side effects. The numerical results show and confirm that the optimal treatment strategies reduce the tumour cells load and increase the effector cells after few days of therapy. The performance of combination therapy protocol was better than the standard protocol of chemotherapy alone. The numerical simulations show the rationality of the model presented, which in some degree meets the natural facts.

This work can be extended to more sophisticated problems with delays in both state and control variables, when the control system is subject to a mixed controlstate constraints.

## Figures and Tables

**Table 1:** The stability results for the coexisting steady states by using the functions ρ and σ, while fixing the rest of the parameters as mentioned in the text.

Region in Figure 2	ρ	σ	Steady state (E*, T*, N*)	Eigenvalues λ_1_, λ_2_, λ_3_	Stability for τ ≥ 0
Light blue (1a)	0.1	0.05	(0.0269, 486.9244, 0.9999)	−0.9509, − 1.9105, −1	Stable node

Light blue (1b)	1.4	0.1	(1.0299, 0.0238, 0.9999)	− 1, − 0.0486 − 0.3096*i*, −0.0486 + 0.3096*i*	Stable spiral for τ < τ*, stable limit cycles at τ = τ*

Orange (2)	0.2	0.23	(0.1648, 419.9672, 0.9999)	−0.5942, − 1, − 1.7876	Stable node

			(0.8656, 79.7712, 0.99)	0.276, − 1, − 0.7291	Unstable saddle node

Brown (3)	0.6	0.1	(0.0789, 461.688, 0.99)	− 0.7137, − 1.5050, − 1	Stable node

			(0.7236, 148.7078, 0.99)	0.4060, − 1, −0.8506	Unstable saddle node

			(1.0298, 0.0623, 0.99)	−1, − 0.0486 − 0.2922*i*, − 0.0486 + 0.2922*i*	Stable spiral for τ < τ*, stable limit cycles at τ = τ*
		

## References

[1] Joshi B, Wang X, Banerjee S, Tian H, Matzavinos A, Chaplain MAJ (2009). On immunotherapies and cancer vaccination protocols: a mathematical modelling approach. *Journal of Theoretical Biology*.

[2] Kirschner D, Panetta JC (1998). Modeling immunotherapy of the tumor—immune interaction. *Journal of Mathematical Biology*.

[3] Kuznetsov VA, Makalkin IA, Taylor MA, Perelson AS (1994). Nonlinear dynamics of immunogenic tumors: parameter estimation and global bifurcation analysis. *Bulletin of Mathematical Biology*.

[4] de Pillis LG, Gu W, Radunskaya AE (2006). Mixed immunotherapy and chemotherapy of tumors: modeling, applications and biological interpretations. *Journal of Theoretical Biology*.

[5] de Pillis LG, Fister KR, Gu W (2008). Optimal control of mixed immunotherapy and chemotherapy of tumors. *Journal of Biological Systems*.

[6] Araujo RP, McElwain DLS (2004). A history of the study of solid tumour growth: the contribution of mathematical modelling. *Bulletin of Mathematical Biology*.

[7] Bellomo N, Li NK, Maini PK (2008). On the foundations of cancer modelling: selected topics, speculations, and perspectives. *Mathematical Models and Methods in Applied Sciences*.

[8] Byrne HM, Alarcon T, Owen MR, Webb SD, Maini PK (2006). Modelling aspects of cancer dynamics: a review. *Philosophical Transactions of the Royal Society of London A*.

[9] Chaplain MAJ (2008). Modelling aspects of cancer growth: insight from mathematical and numerical analysis and computational simulation. *Multiscale Problems in the Life Sciences*.

[10] Martins ML, Ferreira SC, Vilela MJ (2007). Multiscale models for the growth of avascular tumors. *Physics of Life Reviews*.

[11] Nagy JD (2005). The ecology and evolutionary biology of cancer: a review of mathematical models of necrosis and tumor cell diversity. *Mathematical Biosciences and Engineering*.

[12] Roose T, Chapman SJ, Maini PK (2007). Mathematical models of avascular tumor growth. *SIAM Review*.

[13] Yafia R (2006). Dynamics analysis and limit cycle in a delayed model for tumor growth with quiescence. *Nonlinear Analysis: Modelling and Control*.

[14] Yafia R (2011). A study of differential equation modeling malignant tumor cells in competition with immune system. *International Journal of Biomathematics*.

[15] Eftimie R, Bramson JL, Earn DJD (2011). Interactions between the immune system and cancer: a brief review of non-spatial mathematical models. *Bulletin of Mathematical Biology*.

[16] Rihan FA, Rahman DHA (2013). Delay differential model for tumor-immune dynamics with HIV infection of CD4^+^ T-cells. *International Journal of Computer Mathematics*.

[17] Rihan FA, Safan M, Abdeen MA, Abdel Rahman DH (2012). Qualitative and computational analysis of a mathematical model for tumor-immune interactions. *Journal of Applied Mathematics*.

[18] Rihan FA, Abdel Rahman DH, Lakshmanan S, Alkhajeh A (2014). A time delay model of tumour—immune system interactions: global dynamics, parameter estimation, sensitivity analysis. *Applied Mathematics and Computation*.

[19] de Pillis LG, Radunskaya A (2003). The dynamics of an optimally controlled tumor model: a case study. *Mathematical and Computer Modelling*.

[20] Swan GW (1985). Optimal control applications in the chemotherapy of multiple myeloma. *IMA Journal of Mathematics Applied in Medicine and Biology*.

[21] Castiglione F, Piccoli B (2007). Cancer immunotherapy, mathematical modeling and optimal control. *Journal of Theoretical Biology*.

[22] Kirschner D, Tsygvintsev A (2009). On the global dynamics of a model for tumor immunotherapy. *Mathematical Biosciences and Engineering*.

[23] Villasana M, Radunskaya A (2003). A delay differential equation model for tumor growth. *Journal of Mathematical Biology*.

[24] Bodnar M (2000). The nonnegativity of solutions of delay differential equations. *Applied Mathematics Letters. An International Journal of Rapid Publication*.

[25] Halanay A (1966). *Differential Equations: Stability, Oscillations, Time Lags*.

[26] De Pillis LG, Radunskaya A (2001). A mathematical tumor model with immune resistance and drug therapy: an optimal control approach. *Journal of Theoretical Medicine*.

[27] Rihan FA, Doha EH, Hassan MI, Kamel N (2009). Numerical treatments for Volterra delay integro-differential equations. *Computational Methods in Applied Mathematics*.

[28] Baker CTH, Bocharov G, Filiz A Numerical modelling by delay and volterra functional differential equations.

[29] Fleming WH, Rishel RW (1975). *Deterministic and Stocha stic Optimal Control*.

[30] Lukes DL (1982). *Differential Equations: Classical to Controlled*.

[31] Göllmann L, Kern D, Maurer H (2009). Optimal control problems with delays in state and control variables subject to mixed control-state constraints. *Optimal Control Applications & Methods*.

[32] Rihan FA (2000). *Numerical treatment of delay differential equation [Ph.D. thesis]*.

[33] Rihan FA, Rihan BF (2015). Numerical modelling of biological systems with memory using delay differential equations. *Applied Mathematics & Information Sciences*.

